# Improving *bgl1* gene expression in *Saccharomyces cerevisiae* through meiosis in an isogenic triploid

**DOI:** 10.1007/s10529-014-1471-z

**Published:** 2014-02-22

**Authors:** Huajun Yang, Cheng Liu, Shaolan Zou, Yuanyuan Ma, Jiefang Hong, Minhua Zhang

**Affiliations:** 1 School of Chemical Engineering and Technology, Tianjin University, Tianjin, 300072 China; 2 Tianjin R&D Center for Petrochemical Technology, Tianjin University, Tianjin, 300072 China

**Keywords:** β-Glucosidase, δ-Integration, Meiosis, *Saccharomyces cerevisiae*, Sexual spores, Triploid parent

## Abstract

Introducing large numbers of target genes into the chromosome of *Saccharomyces cerevisiae* via δ-sequence-mediated integration is a good strategy for exploring the effects of gene dosage on expression and secretion of heterologous proteins. The expression of exogenous genes might be further improved through meiosis in an isogenic triploid. Here, a stable strain A-8 was screened from 35 sexual spore colonies obtained from an isogenic triploid integratively expressing *bgl1* from *Aspergillus aculeatus*. The corresponding β-glucosidase activity in this strain was increased by ~120 % compared with the parent strain BGL-a. Measurement of doubling time, flow cytometry, and mating experiments further confirmed that A-8 was a spore-forming strain obtained from a triploid parent. Thus, combining δ-integration and meiosis in an isogenic triploid is a promising approach for improving the expression of exogenous proteins in *S. cerevisiae*.

**Electronic supplementary material:**

The online version of this article (doi:10.1007/s10529-014-1471-z) contains supplementary material, which is available to authorized users.

## Introduction

*Saccharomyces cerevisiae* is widely employed for industrial and fuel ethanol production. However, *S. cerevisiae* cannot use cellulose, the most widespread polysaccharide in nature, and therefore genes coding for cellulolytic enzymes are often added to the genome of *S. cerevisiae* to enable cellulose utilization. Despite a number of reports on cellulase genes expression in *S. cerevisiae* (reviewed by Hasunuma and Kondo 2012; la Grange et al. 2010; van Zyl et al. 2007), the expressed enzymatic activities have so far not been sufficient to allow efficient cellulose digestion and strains with increased enzymatic activity are still required.

The expression of exogenous genes in *S. cerevisiae* is influenced by many factors, such as the copy number, the promoter, mitotic stability, mRNA stability and translation efficiency (Ekino et al. 2002). The introduction of large numbers of target genes into the host cells is a widely used approach (Yamada et al. 2010). Traditionally, three types of vectors, YCp, YIp and YEp, have been used for foreign gene expression in *S. cerevisiae*. However, low-copy plasmids, YCp and YIp, and the high-copy but mitotically-unstable plasmid, YEp, make it difficult to assess the effect of gene dosage on expression efficiency. Chromosomal integration via δ-integrative plasmids is a useful method for introducing foreign genes into a yeast cell. These plasmids eliminate cloned gene loss during cell division, allowing structurally-stable insertion of multiple copies of the foreign genes, and the optimum number of genes can be maintained at a constant level (Yamada et al. 2010).

Further increases in the copy number of the integrated genes can be achieved by changing the number of chromosomes. The number of chromosomes may be increased by the duplication of the entire genome, resulting in diploidy or polyploidy, or by the addition of one or more chromosomes, leading to aneuploidy (Torres et al. 2007; Pavelka et al. 2010). While the breeding of polyploidy strains improves enzymatic activity and/or product yield (Ekino et al. 2002; Yamada et al. 2010), aneuploidy has been effective in a wide range of processes and conditions (Jung et al. 2011; Pavelka et al. 2010). The meiotic products derived from a triploid cell would be expected to be highly aneuploid, containing chromosome numbers varying between the haploid number of 16 and the diploid number of 32 (St Charles et al. 2010). Aneuploidy directly impacts gene expression at both the transcriptome and proteome levels and can generate significant phenotypic variation such as cellular growth rate that could bring about fitness gains under diverse conditions (Torres et al. 2007; Jung et al. 2011; Pavelka et al. 2010). In addition, dosage compensation, a process by which genes duplicated by aneuploidy, shows diploid-like expression, as described in plants (Birchler et al. 2005), may occur in these *S. cerevisiae* strains.

β-Glucosidase is an important component synergizing with commercial cellulase. A recombinant haploid *S. cerevisiae* strain expressing β-glucosidase was previously constructed in our laboratory and had the potential for efficient and cost-effective ethanol production from cellulose (Wang et al. 2013). To investigate the possibility of further increasing the expression level by aneuploidy, an isogenic triploid strain integratively expressing the β-glucosidase-encoding gene *bgl1* from *Aspergillus aculeatus* was constructed from a haploid recombinant strain. The resulting spores were purified, isolated, screened, and evaluated for β-glucosidase activity, ploidy, stability, and doubling time. To our knowledge, this is the first study aimed at improving *bgl1* gene expression in *S. cerevisiae* via meiosis in an isogenic triploid.

## Materials and methods

### Strains, media, and cultivation conditions

Table 1 summarizes the genetic properties of all strains used in this study. *S. cerevisiae* strains were cultivated aerobically at 30 °C in YPD medium (20 g peptone/l, 10 g yeast extract/l, and 20 g glucose/l).

**Table 1:** Characteristics of all strains used in this study

Strains	Relevant features	Reference
W303-1A	*MAT*a *leu2-d-3, 112 ura3-1 trp1-92 his3-11, 15 ade2-1 can1-100*	In our lab
BGL-a	*MAT*a *ura3 his3* *trp1 ade can* δ-Integration of *bgl1* gene from *Aspergillus aculeatus*	In our lab
BGL-aα	*MAT*a*/*α *ura3 his3* *trp1 ade can* derivative of BGL-a	This study
BGL-α	*MAT*α *ura3 his3* *trp1 ade can* derivative of BGL-aα	This study
BGL-aa	*MAT*a/a *ura3 his3* *trp1 ade can* derivative of BGL-aα	This study
BGL-aaα	*MAT*a/a/α *ura3 his3* *trp1 ade can* derivative of BGL-aa and BGL-α	This study

### Construction and sporulation of the triploid strain BGL-aaα

Strain BGL-a was constructed by transforming the linearized plasmid pGδL-*bgl1* (Liu et al. 2013) into the haploid *S. cerevisiae* strain W303-1A using the lithium acetate method (Gietz and Sugino 1988), and then the *A. aculeatus* *bgl1* constitutive expression cassette was introduced into the chromosomes of W303-1A via δ-sequence-mediated integration. The diploid strain BGL-aα was constructed by introducing the plasmid YCp50-HO (Herskowitz and Jensen 1991) into BGL-a, and BGL-α was constructed by inducing sporulation in BGL-aα (Hou 2009). Strain BGL-aa was constructed by switching the mating type of BGL-aα using the plasmid YCp33-GHK, as described previously (Hou et al. 2013). The isogenic triploid strain BGL-aaα was constructed by crossing BGL-aa with BGL-α. The schematic diagram for BGL-aaα construction is shown in Supplementary Fig. 1.

Triploid strain BGL-aaα was used to produce spores, as described by Hou (2009). The resulting spores were streaked on YPD plates and cultivated at 30 °C. The mating type of segregants was determined by visual method (Al Safadi et al. 2010). Those strains identified as sexual were further cultivated in YPD medium and used for β-glucosidase activity measurement.

### Assay of enzyme activity

*S. cerevisiae* strains were cultured in YPD medium at 30 °C for 3 days. Cultures were then tested for β-glucosidase activity using a method described by Liu et al. (2013). One unit of activity was defined as the amount of enzyme that liberated 1 μmol *p*-nitrophenol per min under the assay conditions.

### Ploidy, stability and doubling time determination

*S. cerevisiae* strains were cultivated aerobically at 30 °C in YPD medium. For ploidy determination, stationary-phase cells were used to determine the DNA content using flow cytometory analysis (FCAS), as described by Carlson et al. (1997). For stability measurement, *S. cerevisiae* strains were cultured to stationary phase, and then inoculated into fresh medium at 1 % (v/v). This process was repeated ten times. One transfer implies at least five generation times, thus the final generation times were greater than 50. The β-glucosidase activity of these cultures was then measured to evaluate the activity stability. For determination of doubling time, samples were taken at 2 h intervals for OD_600_ measurements.

## Results

### Construction of triploid strain BGL-aaα and the resultant spore strains selection by β-glucosidase activity

Our previous work showed that δ-integration is a good method for obtaining recombinant strains with multiple copy number and stable expression of exogenous genes in *S. cerevisiae* (Liu et al. 2013; Wang et al. 2013), and the defective *leu2*-*d* allele used as the selection marker for the δ-integrative plasmid could further increase the copy number as well as the enzymatic activity (Liu et al. 2013). Therefore, the haploid strain BGL-a was constructed by introducing a *bgl1* gene expression cassette into W303-1A via a δ-integrative plasmid using the *LEU2*-*d* selective marker. The β-glucosidase activity of the resulting strain BGL-a was 15.7 ± 0.76 U/ml.

Although several rounds of δ-integration could be preformed, many rounds of retransformation were unproductive (Lee and Da Silva 1997). δ-Integration occurs on multiple chromosomes due to the presence of about 425 δ-sequences dispersed throughout the yeast genome (Dujon 1996). Meiosis in triploid would result in four highly aneuploid gametes because six copies of each homolog must be segregated into four meiotic products (St Charles et al. 2010). Those gametes may or may not be viable (St Charles et al. 2010; Thorburn et al. 2013; Torres et al. 2007). Thus, it is possible to screen the viable gametes to identify a strain with extra chromosomes and many copies of the cloned gene(s).

Based on the aforementioned speculation, BGL-a was selected as the parent strain to construct triploid strain BGL-aaα (Table 1 and Supplementary Fig. 1). To obtain meiotic products of strain BGL-aaα, tetrad dissection was performed. Only five spores grew and formed colonies on YPD plates from a total of ten sporangia, so the viability of these spores was as low as 12.5 %. Interestingly, all five gametes were sexual using a mating type test, with four capable of mating with BGL-α (*MAT*α), and one successfully mating with W303-1A (*MAT*a). Thus, directly purifying and isolating the spores on YPD plates followed by the mating type testing to screen out sexual strains was used instead of the tetrad dissection method.

Using this method, 35 viable sexual strains were selected from 65 spore colonies, of which 18 strains were from large colonies and 17 were from small colonies. Nine of the strains could mate with W303-1A (*MAT*a), while the remaining 26 could mate with BGL-α (*MAT*α) (Supplementary Table 1). Activity measurement (Supplementary Table 1) showed that: (1) all 35 strains had highly variable BGL activity ranging from 34 ± 1.3 to 3.1 ± 0.11 U/ml, especially in strains from large colonies; (2) 17 strains showed higher β-glucosidase activity than the parent strain BGL-a (15.7 ± 0.76 U/ml), of which eight strains were from big colonies and nine were from small colonies; (3) four strains, A-8, A-30, A-41, and A-51, had particularly high activity, showing increases of 117, 61, 84, and 111 %, respectively, compared with BGL-a. The possible relationship between colony size and the mating type or enzymatic characteristics is unclear and warrants further investigation.

### Ploidy, stability, and doubling time determination

**Fig. 1:**
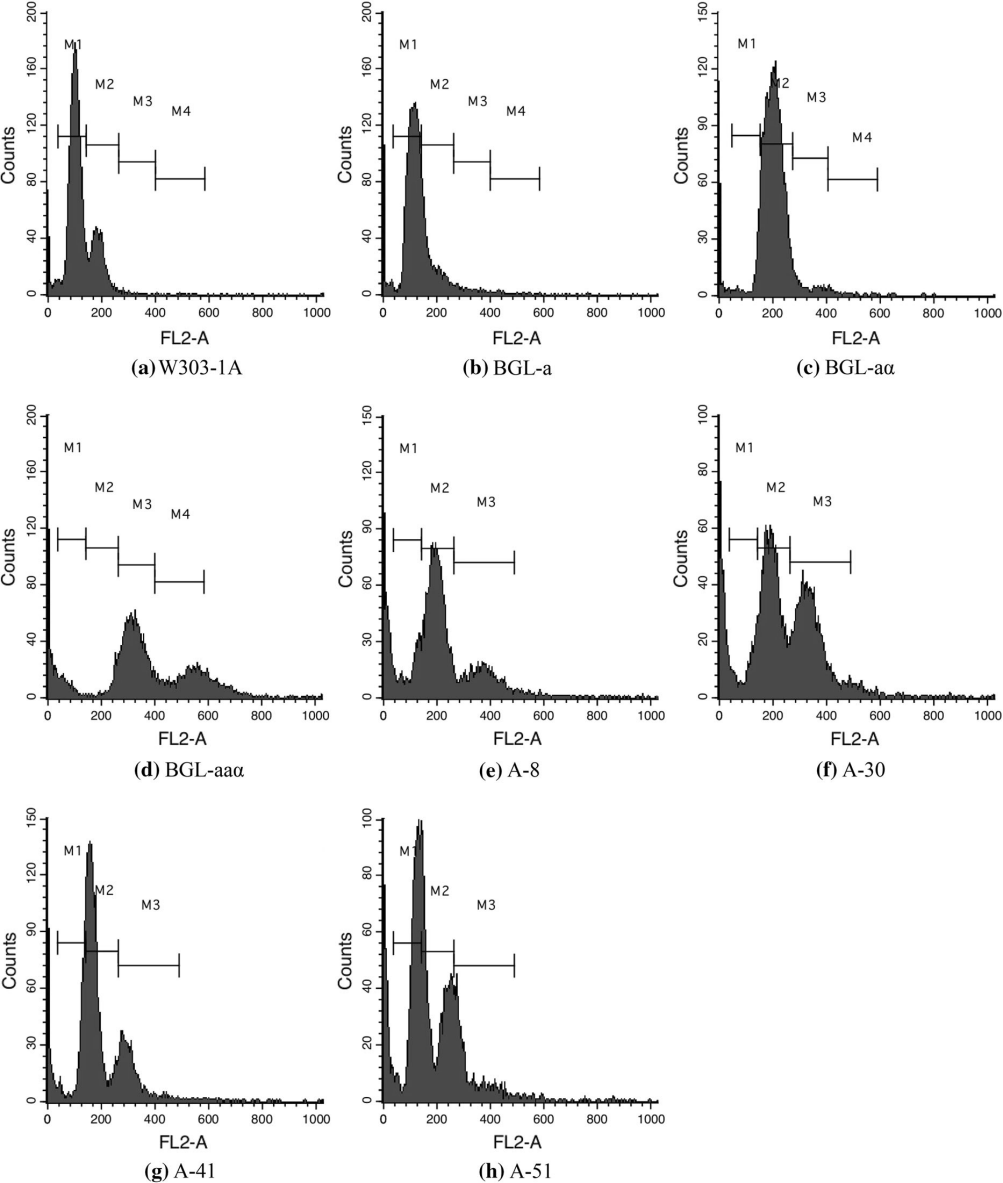
FCAS of *S. cerevisiae* strains

To determine whether the four high expression strains, A-8, A-30, A-41, and A-51, were aneuploid yeast strains as expected, their DNA content was determined by FCAS. The results are shown in Fig. 1. While the results for BGL-a, BGL-aα, and BGL-aaα showed that propidium iodide staining was linearly related to ploidy, the four high expression strains exhibited a transition state between haploid and diploid, which was consistent with a previous study (Al Safadi et al. 2010).

**Fig. 2:**
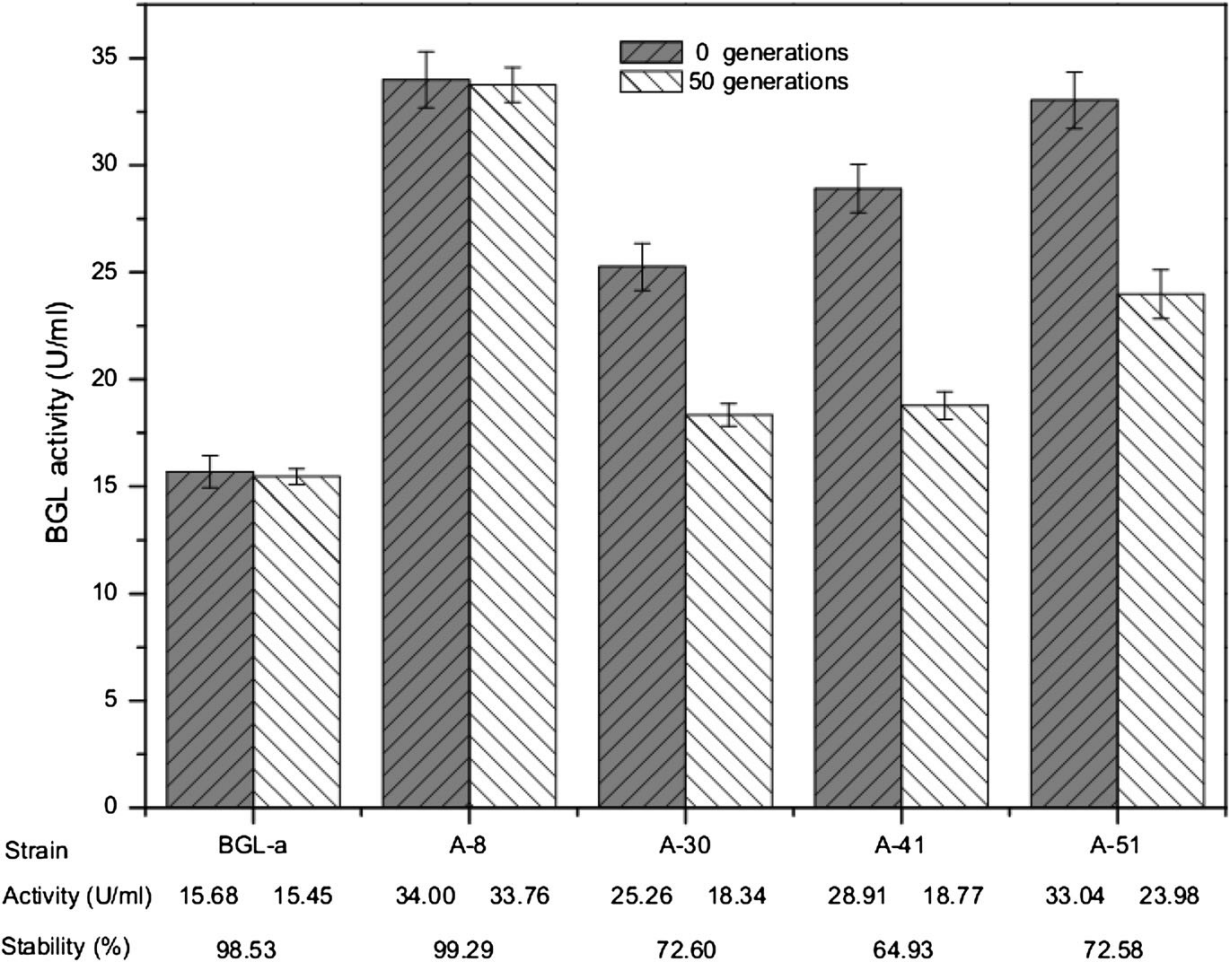
The BGL activities and stabilities of *S. cerevisiae* strains

The stability of exogenous gene expression is important for industrial applications, especially for the aneuploid strains, which are generally thought to be unstable. We therefore tested the stability of the activity of the high expression strains and the parent BGL-a by serial cultivation in YPD medium (Fig. 2). The results showed that the gene expression in strain A-8 was particularly stable. To examine the effect of meiosis on activity stability, we also tested the remaining 13 strains with higher activity than that of BGL-a. The results showed that two strains, A-12 and A-43, also remained relatively stable, and their expression stabilities were maintained over 90 %.

**Fig. 3:**
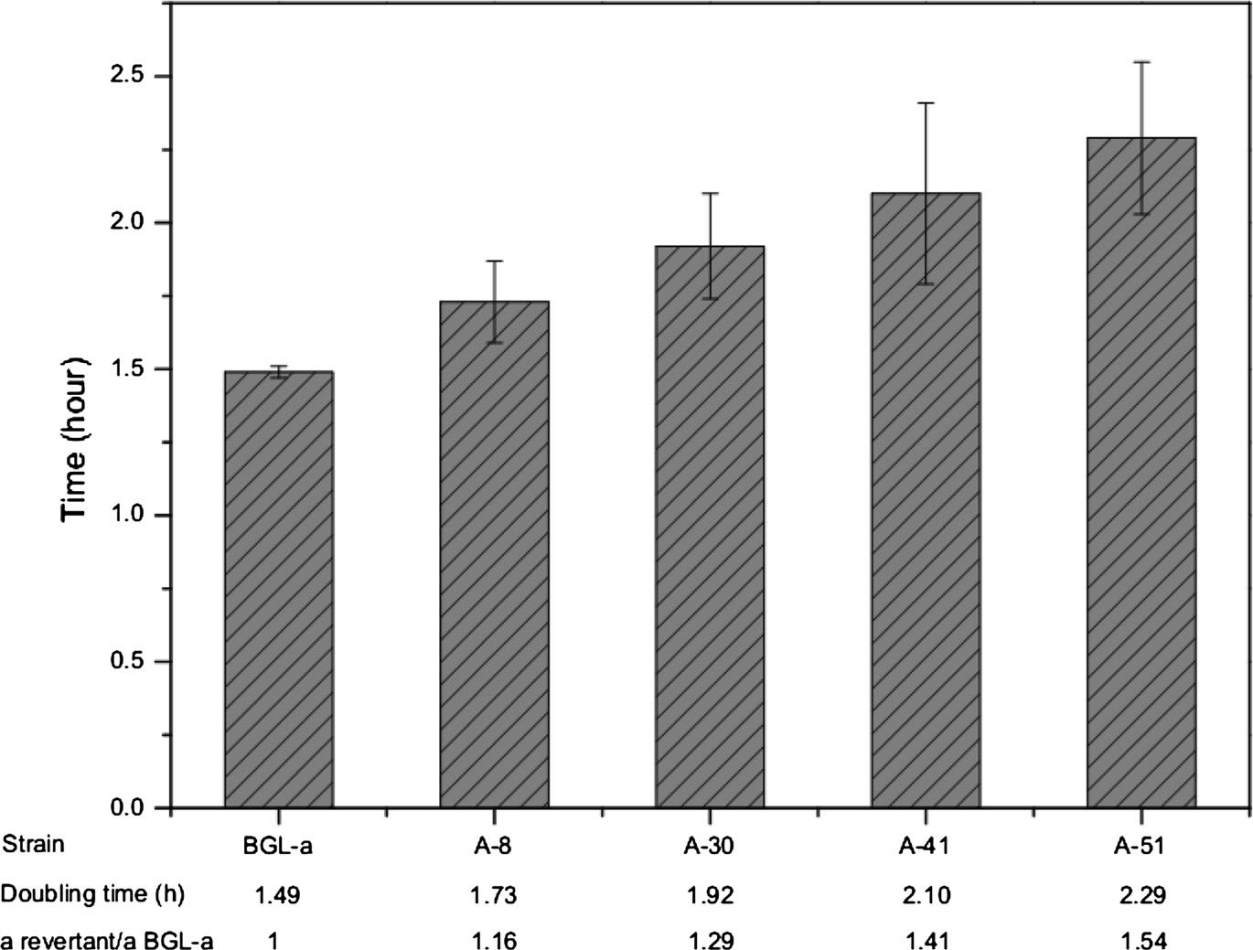
Doubling time of *S. cerevisiae* strains

Aneuploid yeast strains exhibit defects in cell growth, and doubling time can be used as a measure of growth ability. Therefore, we examined the doubling time of the high expression strains and the parent strain BGL-a (Fig. 3). Strains A-8, A-30, A-41, and A-51 all had longer doubling times than BGL-a, BGL-aα and BGL-aaα, which were 1.49 ± 0.02, 1.48 ± 0.03, and 1.52 ± 0.05 h, respectively.

The above results of ploidy, stability, and doubling time assays, along with the mating ability, indicated that the four high expression strains, A-8, A-30, A-41, and A-51, were spore-forming strains that arose as meiotic products from the isogenic triploid BGL-aaα.

## Discussion

Aneuploidy is associated with developmental defects, cancer, and adaptive evolution in experimental organisms (Thorburn et al. 2013; Torres et al. 2007; Pavelka et al. 2010). Although it remains unresolved as to how aneuploidy impacts gene expression (Pavelka et al. 2010), our study indicates the potential of aneuploidy for further improving enzymatic activity and fitness of *S. cerevisiae*. The strain with the highest level of expression, A-8, which was the meiotic product of isogenic triploid strain BGL-aaα, not only had higher activity than that of euploid counterparts BGL-a, BGL-aα and BGL-aaα (data not shown), but also exhibited stable foreign gene expression. This possibly contributed to increased gene dosage and adaptive evolution (Pavelka et al. 2010), or a balance phenomenon, such that changes in individual chromosomal dosage altered the phenotype more dramatically than changes in ploidy (Birchler et al. 2005).

The efficiency of protein secretion in yeast is affected by how the heterologous gene is maintained in the cell. A previous study showed that when a gene is fused to a yeast promoter and the secretion signal sequence was integrated into a yeast chromosome, a greater proportion of the protein was secreted than when the same construct was introduced on a multicopy plasmid vector (Smith et al. 1985). Thus, introducing exogenous genes into a yeast cell by chromosomal integration was preferred (Ekino et al. 2002). Multicopy δ-integration of target genes is also more effective for improving enzyme activity than the integrative but low-copy plasmid YIp, although it was not proportionate (Yamada et al. 2010; Liu et al. 2013). On the other hand, the expression of the δ-sequence was shown to be governed by haploid-specific transcriptional activation, the expression level of a δ-integrated exogenous gene in a a/α diploid cell would be much lower than that in a haploid cell (Ekino et al. 2002). This is another possible cause of the observed higher activity of strain A-8 compared with that of BGL-aα and BGL-aaα (data not shown), as strain A-8 was shown to be haploid-like *MAT*α, while the latter strains were both *MAT*a/α.

Therefore, δ-integration is a good strategy for exploring the effects of gene dosage on expression and secretion of heterologous proteins in *S. cerevisiae*. It is possible to further increase the expression level of the integrated genes by changing the number of chromosomes through meiosis in an isogenic triploid. In this study, the stable strain A-8, integratively expressing *bgl1* from *A. aculeatus*, was obtained using a sporulating triploid strain. The β-glucosidase activity of A-8 increased by 116.84 % compared with parent strain BGL-a, though its doubling time was slightly longer than that of the parent. Our results suggest that combining δ-integration and meiosis in an isogenic triploid is a promising approach for improving the expression of exogenous proteins in *S. cerevisiae*.

## Electronic supplementary material

Below is the link to the electronic supplementary material.

Supplementary material 1 (DOCX 70 kb)
