# Supplementary material for: Improving bgl1 gene expression in Saccharomyces cerevisiae through meiosis in an isogenic triploid
Source: Biotechnol Lett. 2014 Feb 22;36(6):1279–85. doi: 10.1007/s10529-014-1471-z (PMC4000627; doi:10.1007/s10529-014-1471-z)
Supplement: Supplementary file 1 — Supplementary material 1 (DOCX 70 kb) [file 10529_2014_1471_MOESM1_ESM.docx]

**Supplementary Fig. 1** The schematic diagram for the triploid strain BGL-aaα construction

**Supplementary Table 1** Characteristics of all sexual strains

| Strains | Colony size | Mate with MATa strain | Mate with MATα strain | β-Glucosidase activities (U/ml) |
| --- | --- | --- | --- | --- |
| A-8 | Big | + | - | 34.00 ± 1.31 |
| A-30 | Big | - | + | 25.26 ± 1.10 |
| A-4 | Big | - | + | 21.84 ± 0.92 |
| A-6 | Big | - | + | 20.88 ± 0.87 |
| A-13 | Big | - | + | 19.87 ± 0.87 |
| A-10 | Big | - | + | 18.56 ± 0.86 |
| A-12 | Big | - | + | 17.23 ± 0.81 |
| A-37 | Big | + | - | 15.89 ± 0.72 |
| A-16 | Big | - | + | 14.64 ± 0.66 |
| A-7 | Big | - | + | 14.34 ± 0.65 |
| A-31 | Big | - | + | 13.59 ± 0.61 |
| A-40 | Big | - | + | 13.22 ± 0.62 |
| A-28 | Big | - | + | 12.57 ± 0.58 |
| A-3 | Big | - | + | 11.98 ± 0.55 |
| A-33 | Big | + | - | 10.70 ± 0.49 |
| A-35 | Big | - | + | 9.18 ± 0.41 |
| A-11 | Big | + | - | 4.21 ± 0.22 |
| A-38 | Big | - | + | 3.14 ± 0.11 |
| A-51 | Small | - | + | 33.04 ± 1.32 |
| A-41 | Small | - | + | 28.91 ± 1.13 |
| A-48 | Small | - | + | 22.68 ± 1.02 |
| A-43 | Small | - | + | 21.54 ± 0.93 |
| A-63 | Small | - | + | 19.01 ± 0.88 |
| A-55 | Small | - | + | 17.06 ± 0.77 |
| A-64 | Small | - | + | 16.78 ± 0.74 |
| A-44 | Small | + | - | 15.89 ± 0.75 |
| A-60 | Small | + | - | 15.80 ± 0.71 |
| A-53 | Small | - | + | 15.64 ± 0.65 |
| A-58 | Small | - | + | 13.78 ± 0.53 |
| A-57 | Small | - | + | 13.76 ± 0.62 |
| A-47 | Small | - | + | 13.37 ± 0.54 |
| A-62 | Small | - | + | 13.31 ± 0.64 |
| A-54 | Small | + | - | 13.20 ± 0.58 |
| A-45 | Small | + | - | 12.25 ± 0.59 |
| A-56 | Small | + | - | 10.65 ± 0.48 |
